# Patient-Reported Outcome, Return to Sport, and Revision Rates 7-9 Years After Anterior Cruciate Ligament Reconstruction: Results From a Cohort of 2042 Patients

**DOI:** 10.1177/03635465211060333

**Published:** 2022-01-18

**Authors:** Per-Henrik Randsborg, Nicholas Cepeda, Dakota Adamec, Scott A. Rodeo, Anil Ranawat, Andrew D. Pearle

**Affiliations:** † ACL Study Group, Sports Medicine Institute, Hospital for Special Surgery, New York, New York, USA; ‡ Akershus University Hospital, Department of Orthopedic Surgery, Lørenskog, Norway; Investigation performed at the Hospital for Special Surgery, New York, New York, USA

**Keywords:** ACL, IKDC, return to sport, revision

## Abstract

**Background:**

Long-term patient-reported outcome measures (PROMs), rates of return to sport, and revision risk after anterior cruciate ligament (ACL) reconstruction (ACLR) are not well understood.

**Purpose:**

To provide long-term follow-up of PROMs, return-to-sport rates, and revision rates after ACLR and to identify predictors for poor outcome.

**Study Design:**

Case-control study; Level of evidence, 3.

**Methods:**

A total of 2042 patients were included in an institutional ACL registry (2009-2013) and longitudinally followed. PROMs were completed preoperatively and at all follow-up time points. Questions regarding return to sport and knee stability were completed at final follow-up. Predictors for poor outcome on the International Knee Documentation Committee (IKDC) score were estimated in a regression model incorporating risk factors such as patient characteristics, graft choice, and concomitant injuries. Revision rates and risk of subsequent non-ACL surgeries were calculated.

**Results:**

Autografts were used in 76% of the patients (patellar tendon, 62%; hamstring grafts, 38%). Allografts were used in 24% of patients. The questionnaires were returned by 1045 (51.2%) patients at a mean of 7.2 years (range, 5.0-9.8 years) after surgery. Improvements in IKDC score of >30 points were sustained for all patient categories. The strongest predictor for lesser improvement in IKDC score was a cartilage lesion >2 cm^2^ identified during surgery. Male sex and college education completion were associated with improved IKDC scores. Meniscal lesions did not predict change) in the IKDC score. A total of 69% of patients had returned to sport after 8.1 years (range, 6.7-9.8 years). The main reason for not returning to sport was fear of reinjury. The revision rate was 7.2% after 9 years (range, 8-11 years), 13% of patients needed subsequent ipsilateral non-ACL surgery, and 6% underwent contralateral ACLR. The absence of a meniscal tear, younger age, and male sex were predictors for revision. Graft choice did not predict PROM results or revision risk.

**Conclusion:**

Improvements in IKDC scores were sustained 7 years after ACLR. The strongest predictor for poor outcome was a cartilage lesion >2 cm^2^. Patients can expect a 70% return-to-sport rate and an 87% chance of their knee feeling stable during daily and athletic activities after 8 years. Young male patients have better PROM scores but a higher risk of revision. There is a 26% chance of subsequent knee surgery within 9 years, including a revision rate of 7%, subsequent non-ACL surgery to the operated knee in 13%, and a 6% chance of contralateral ACLR.

A rupture of the anterior cruciate ligament (ACL) is a common injury that can lead to instability and inability to partake in sports activities.^3^ ACL reconstruction (ACLR) is therefore recommended in patients who wish to return to pivoting sports.^28^ ACLR is one of the most commonly performed surgical procedures in athletes, with high rates of return to sport and patient satisfaction.^22^ However, graft failure, the need for revision ACLR, contralateral ACL rupture, and subsequent knee injuries such as cartilage and meniscal lesions are well documented.^1,2^

The safety and success of ACLR are often evaluated by the revision rate. However, this is an incomplete measure of the success of ACLR. Some patients with graft failures might cope with occasional instability and not seek revision surgery, whereas other patients might have an intact graft but experience complications such as pain, stiffness, or even instability. Therefore, patient-reported outcome measures (PROMs) are important tools to evaluate the effectiveness of ACLR. ACLR is a significant intervention, and the physical, mental, and neuromuscular rehabilitation can take 18 months or more.^21^ Two-year follow-up may not provide insight into the longer term success of the procedure. Furthermore, the loss to follow-up is often considerable. For example, the loss to follow-up at 2 years in the Scandinavian registries is about 50%.^13,25^ The Kaiser Permanente Registry presented 5-year PROM results, but the response rate was only 23%.^4^ Most ACL registries provide 1- and 2-year PROM data, whereas longer term follow-up data of larger cohorts are scant. A systematic review could identify only 7 studies, with a mean sample size of 59 patients, that presented 10-year PROM data.^5^ Aggregate data analysis was hampered by heterogeneity and small sample size. In another systematic review, Magnussen et al^15^ included 13 studies with a minimum follow-up of 10 years and a mean sample size of 268 patients. Only 1 study included >1000 patients. The authors stated, “Further large prospective cohort studies with good follow-up, consistent outcome reporting, and regression modeling are needed to clarify predictors of long-term patient-reported outcome of ACL reconstruction.”^15^

The selection bias is further exaggerated by the fact that more female patients and older patients return the questionnaires.^10^ The Multicenter Orthopaedic Outcomes Network (MOON) is the only cohort with long-term follow-up of PROM data with high compliance.^19^ The investigators included 1592 patients between 2002 and 2004 and reported 83% follow-up at 10 years.

The 2-year results from the Hospital for Special Surgery (HSS) ACL Registry have previously been described, providing information on patient characteristics, graft choice, early revision rates, and PROM results.^17^ The purpose of the current study is to provide a mid- to long-term follow-up of the same cohort, focusing on PROMs, return-to-sport rates, and revision rates after primary ACLR, and to identify predictors for poor outcome.

## Methods

### Participants

The HSS ACL Registry was established for the purpose of monitoring and improving the quality of ACLR performed at HSS. All patients of all ages scheduled for a primary ACLR with or without concomitant procedures or previous ACLR to the contralateral knee were eligible for inclusion.

### Data Collection

The participating surgeons provided clinical data and intraoperative findings at the time of surgery, such as graft choice and details about concurrent meniscal and cartilage lesions. The patients completed the following validated PROMs at baseline (preoperatively): the International Knee Documentation Committee (IKDC) Subjective Knee Evaluation, Lysholm score, Tegner scale, Marx activity score, and 12-Item Short Form Health Survey. These were completed again after 2 and 5 years. Finally, patients were invited for a follow-up in 2020 to complete the IKDC evaluation and Marx activity score and respond to specific study questions regarding return to sport and knee stability.

The medical records of the institution were searched by procedure codes in January 2021 to identify patients who had returned for any type of knee surgery after their primary ACLR, including ACLR revision and ACLR in the contralateral knee. Risk of revision and subsequent non-ACL knee surgeries were estimated based on data from the medical files and self-reported knee surgery history.

### Statistical Analysis

Continuous variables are presented as mean with 95% CI and/or standard deviation. Categorical variables are presented as frequencies (percentage). Groups were compared using Student *t* test or Pearson chi-square test. The changes in PROM scores from baseline to final follow-up were evaluated by crude (unstratified) analysis. A stepwise multivariable linear regression model analyzed the association between predictor variables (risk factors) and change in IKDC scores from baseline to final follow-up. To identify possible risk factors for revision, a multivariate logistic regression analysis was performed. Baseline (preoperative) variables considered for inclusion in the regression models were selected based on a combination of univariate analysis, known risk factors from the literature, and clinical experience. The patient characteristics considered were age at the time of surgery, sex, ethnicity, body mass index, level of education, smoking status, baseline activity level, and injury mechanism. Surgical risk factors considered for inclusion in the regression models were graft type, meniscal tears discovered at the index surgery (classified as medial, lateral, or both), treatment of meniscal injury (meniscectomy, repair, or no treatment), and cartilage injury identified at the time of surgery (categorized as lesion size smaller or larger than 2 cm^2^). Further clinical risk factors were knee laxity determined during examination under anesthesia (pivot shift, Lachman test) and coronal instability (medial collateral ligament and lateral collateral ligament deemed loose or stable at 30° of knee flexion). *P* values <.05 were considered statistically significant. Statistical analyses were performed with SPSS for Windows (Version 26; IBM Corp).

### Ethics

The study was conducted in accordance with the Declaration of Helsinki Ethical Principles for Medical Research Involving Human Subjects. The study was approved by the institutional review board (IRB No. 2013-018). All patients provided written informed consent.

## Results

### Baseline Data

**Figure 1.**
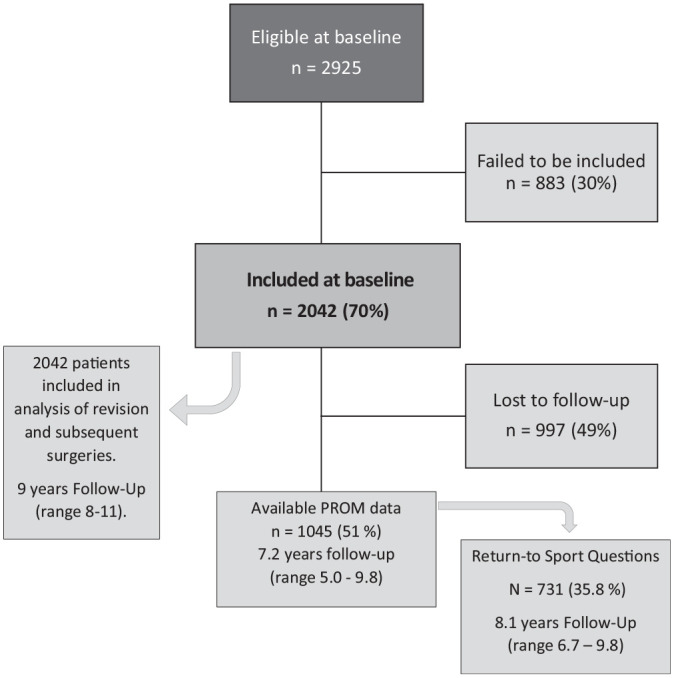
Flowchart of included patients. PROM, patient-reported outcome measure.

Between June 1, 2009, and September 6, 2013, a total of 2925 primary ACLRs were performed, of which 2042 (70%) were primary unilateral ACLRs and were included in the study (Figure 1). The patient characteristics and concomitant injuries are presented in Table 1. There were 1172 (57.4%) male patients, and the mean age at the time of surgery was 29.7 ± 11.95 years. The ACLR was performed with an autograft in 1295 of 1695 patients (76.4%) with available data. A concomitant cartilage lesion was identified in 415 (20.3%) patients. A meniscal injury at the time of surgery was identified in 1018 (49.9%) patients. The most common treatment for meniscal injury was meniscectomy, being performed in 663 (53.6%) patients with meniscal tears, whereas 446 (36.0%) meniscal lesions were repaired. The remaining 132 (10.7%) meniscal lesions were untreated. An isolated lateral meniscal tear was the most common meniscal injury, found in 417 (20.3%) knees (Table 2).

**Table 1:** Baseline Characteristics, Concomitant Injuries, and Graft Choice in 2042 Patients Undergoing Primary Anterior Cruciate Ligament Reconstruction

Variable	n (%) or Mean ± SD
Age at surgery, y (N = 2042)	29.7 ± 11.95
Female	870 (42.6)
Male	1172 (57.4)
Body mass index (n = 1897)	24.7 ± 3.9 (range, 15.7-45.5)
Ethnicity (N = 2042)	
White	1464 (71.7)
Asian or Pacific Islander	168 (8.2)
Hispanic	122 (6.0)
Black	78 (3.8)
Native American Indian	20 (1.0)
Other or unspecified	190 (9.3)
Smoking status (n = 1920)	
Never smoked	1565 (81.5)
Previous smoker	248 (12.9)
Current smoker	107 (5.6)
Level of education (n = 1924)	
High school or less	470 (24.4)
College	946 (49.2)
Postgraduate	508 (26.4)
Sport played at the time of injury (n = 1554)	
Skiing	414 (26.6)
Soccer	311 (20.1)
Basketball	223 (14.4)
Football	111 (7.1)
Lacrosse	96 (6.2)
Tennis	38 (2.4)
Martial arts	31 (2.0)
Dancing	25 (1.6)
Snowboarding	19 (1.2)
Volleyball	18 (1.2)
Running	11 (0.7)
Cycling	10 (0.6)
Wrestling	10 (0.6)
Other	237 (15.3)
Mechanism of injury (n = 1615)	
Contact	294 (18.2)
Noncontact	1321 (81.8)
Level of activity (n = 1595)	
Recreational	1138 (71.3)
High school	300 (18.8)
College	135 (8.5)
Professional	22 (1.4)
Meniscal injuries (N = 2042)	
No	1024 (50.1)
Yes	1018 (49.9)
Lateral	417 (20.4)
Medial	360 (17.7)
Medial and lateral	241 (11.8)
Cartilage injuries (N = 2042)	
No	1627 (79.7)
Yes	415 (20.3)
Graft choice (n = 1695)	
Autograft	1295 (76.4)
Bone–patellar tendon–bone	797 (61.5)
Hamstring	496 (38.3)
Quadriceps	2 (0.2)
Allograft	400 (23.6)
Achilles	312 (78.0)
Tibialis anterior	31 (7.8)
Hamstring	31 (7.8)
Tibialis posterior	15 (3.8)
Quadriceps bone	7 (1.8)
Unspecified	4 (1.0)

**Table 2:** Treatment of 1238 Meniscal Tears in 1018 Patients^*a*^

	Meniscectomy	Repair	No Treatment
Overall (n = 1238)	663 (53.6)	446 (36.0)	129 (10.4)
Lateral meniscus (n = 417)	243 (58.3)	115 (27.6)	59 (14.1)
Medial meniscus (n = 360)	177 (49.2)	160 (44.4)	23 (6.4)
Both menisci (n = 482)^*b*^	243 (50.4)	171 (35.5)	50 (10.4)

a Data are expressed as n (%).

b Treatment type is missing for 1 of the menisci in 21 of 241 patients with both lateral and medial tears.

### Patient-Reported Outcome

PROM data were available for 1045 (51.2%) patients, after a mean follow-up of 7.2 years (range, 5.0-9.8 years). Improvements in IKDC scores were sustained, with increases from baseline of >30 points for all patient categories (Table 3). Patients with a concomitant meniscal injury had a statistically significant lower IKDC score at baseline (*P* < .001) compared with patients without a meniscal lesion, and the improvement in IKDC from baseline to last follow-up was statistically significantly larger for patients with meniscal pathology (34.8 [95% CI, 33.0-36.5] for patients with a meniscal lesion vs 31.3 [95% CI, 29.5-33.1] for patients without a meniscal lesion; *P* = .007). At final follow-up, there was no difference in IKDC score between patients with or without meniscal injury. Patients with cartilage injuries had lower Marx activity score compared with patients without cartilage injuries (10.5 vs 11.7, respectively; *P* < .001). However, patients without cartilage lesions had a larger decline in activity score (*P* = .02), so that at final follow-up there was no difference between the groups (7.6 vs 7.7, respectively; *P* = .8). When controlling for likely confounding variables in a multivariate linear regression analysis, we found no differences in IKDC and Marx scores at final follow-up between patients with or without meniscal and cartilage injuries (Table 4). However, patients with cartilage lesions >2 cm^2^ (n = 37) had a clinically meaningful and statistically significant lower IKDC score at final follow-up compared with patients with a cartilage lesion <2 cm^2^ (69.22 [95% CI, 60.3-78.1] vs 84.18 [95% CI, 81.3-87.1], respectively; *P* = .004).

**Table 3:** Crude Mean IKDC and Marx Activity Scores at Baseline (Preoperatively) and at Final Follow-up at a Mean of 7.2 Years (Range, 5.0-9.7 Years)^*a*^

	Baseline		Last Follow-up		Change
	n	Mean (95% CI)	n	Mean (95% CI)	Mean (95% CI)
IKDC score					
All patients	2042	51.8 (51.1 to 52.4)	1045	84.2 (83.3 to 85.1)	33.0 (31.7 to 34.2)
Age ≤18 y	462	53.8 (52.4 to 55.2)	196	84.1 (81.9 to 86.2)	31.2 (28.3 to 34.1)
Age 19-25 y	432	52.2 (50.7 to 53.7)	200	84.1 (81.9 to 86.2)	31.5 (28.3 to 34.6)
Age 26-30 y	286	50.7 (48.9 to 52.5)	163	84.0 (81.8 to 86.2)	33.8 (30.8 to 36.7)
Age >30 y	861	50.8 (49.7 to 51.9)	486	84.4 (83.2 to 85.6)	34.1 (32.3 to 35.9)
Female	870	51.5 (50.4 to 52.5)	458	83.4 (82.1 to 84.8)	32.3 (30.2 to 34.1)
Male	1172	52.0 (51.1 to 52.9)	587	84.8 (83.6 to 86.0)	33.6 (32.0 to 35.3)
Autograft	1295	52.5 (51.6 to 53.3)	636	84.7 (83.6 to 85.9)	32.9 (31.4 to 34.5)
BTB	797	52.1 (51.0 to 53.2)	394	84.6 (83.1 to 86.1)	33.0 (30.9 to 35.0)
Hamstring	496	53.1 (51.7 to 54.5)	242	84.9 (83.2 to 86.7)	32.9 (30.3 to 35.5)
Allograft	400	49.6 (48.0 to 51.2)	236	82.9 (81.1 to 84.6)	34.4 (31.7 to 37.0)
Meniscal lesion	1018	50.6 (49.6 to 51.6)	509	84.1 (82.8 to 85.3)	34.8 (33.0 to 36.5)
No meniscal lesion	1024	52.9 (51.9 to 53.9)	536	84.3 (83.1 to 85.5)	31.3 (29.5 to 33.1)
Cartilage lesion	415	51.1 (49.5 to 52.7)	277	83.8 (81.8 to 85.8)	34.7 (31.8 to 37.5)
No cartilage lesion	1627	51.9 (51.1 to 52.7)	818	84.3 (83.4 to 85.3)	32.5 (31.1 to 33.9)
Marx activity score					
All patients	2042	11.5 (11.2 to 11.7)	1038	7.7 (7.4 to 8.0)	−3.6 (−4.0 to −3.2)
Age ≤18 y	462	14.4 (14.1 to 14.7)	194	8.6 (7.9 to 8.3)	−5.8 (−6.6 to −5.0)
Age 19-25 y	432	12.2 (11.8 to 12.7)	200	8.2 (7.6 to 8.8)	−3.8 (−4.6 to −2.9)
Age 26-30 y	286	10.9 (10.4 to 11.4)	161	7.3 (6.6 to 8.0)	−3.5 (−4.4 to −2.5)
Age >30 y	861	9.7 (9.3 to 10.0)	483	7.3 (6.9 to 7.7)	−2.6 (−3.2 to −2.1)
Female	869	11.5 (11.2 to 11.8)	452	7.6 (7.2 to 8.1)	−3.9 (−4.4 to −3.3)
Male	1172	11.4 (11.1 to 11. 7)	586	7.8 (7.4 to 8.2)	−3.4 (−3.8 to −2.9)
Autograft	1295	12.2 (11.9 to 12.4)	630	8.2 (7.8 to 8.5)	−4.0 (−4.4 to −3.5)
BTB	797	12.7 (12.4 to 13.0)	392	8.2 (7.8 to 8.7)	−4.5 (−5.1 to −3.8)
Hamstring	496	11.3 (10.9 to 11.7)	238	8.0 (7.4 to 8.6)	−3.1 (−3.8 to −2.4)
Allograft	400	9.5 (9.0 to 10.0)	235	6.9 (6.3 to 7.5)	−2.6 (−3.3 to −1.8)
Meniscal lesion	1018	11.5 (11.2 to 11.8)	507	7.9 (7.5 to 8.4)	−3.5 (−4.0 to −3.0)
No meniscal lesion	1024	11.4 (11.1 to 11.7)	531	7.5 (7.1 to 7.9)	−3.7 (−4.2 to −3.2)
Cartilage lesion	415	10.5 (10.0 to 11.0)	225	7.6 (7.0 to 8.3)	−2.8 (−3.5 to −2.0)
No cartilage lesion	1627	11.7 (11.5 to 11.9)	813	7.7 (7.4 to 8.1)	−3.8 (−4.2 to −3.4)

a BTB, bone–patellar tendon–bone; IKDC, International Knee Documentation Committee.

**Table 4:** Regression Analysis of the Association Between Meniscal and Cartilage Lesions Identified at the Time of Surgery and IKDC and Marx Scores Reported 7.2 Years (Range, 5.0-9.7 Years) Later^*a*^

	Meniscal Lesions^*b*^			Cartilage Lesions^*c*^		
	β	95% CI	*P* Value	β	95% CI	*P* Value
IKDC						
Unadjusted	−0.24	−2.0 to 1.5	.79	−0.56	−2.69 to 1.56	.60
Adjusted	−0.43	−2.47 to 1.61	.68	−0.19	−2.62 to 2.25	.88
Marx						
Unadjusted	0.43	−0.15 to 1.0	.15	−1.0	−0.79 to 0.59	.78
Adjusted	0.42	−0.25 to 1.09	.22	0.25	−0.55 to 1.06	.54

a Adjusted for age, sex, body mass index, ethnicity, smoking status, education level, and graft choice. IKDC, International Knee Documentation Committee subjective knee evaluation form.

b No meniscal lesion used as reference.

c No cartilage lesion used as reference.

Patients receiving autografts were younger and had a higher baseline Marx activity score than allograft recipients. The difference in Marx activity score remained statistically significant at final follow-up but had become smaller.

Predictors for improvement in IKDC score from baseline to final follow-up are presented in univariate analysis (Appendix Table A1, available in the online version of this article) and multivariate regression analysis (Table 5). The strongest predictor for lesser improvement in IKDC score was having a cartilage lesion >2 cm^2^. Male sex and having completed a college education were positive predictors for improvement in IKDC score.

**Table 5:** Multivariate Linear Regression Analysis of Predictors (Positive or Negative) for Improvement in IKDC Score From Baseline to Final Follow-up^*a*^

	Coefficient	95% CI	*P* Value
Cartilage lesion >2 cm^2^	−13.74	−23.25 to −4.24	.005
Higher education^*b*^	5.50	2.12 to 8.89	.001
Smoking	−2.98	−6.29 to 0.33	.077
Baseline Marx score	0.32	0.08 to 0.55	.009
Baseline IKDC score	−0.94	−1.00 to 0.89	<.001
Female sex	−2.16	−4.06 to 0.26	.026
Age	−0.11	−0.23 to 0.004	.07

a Adjusted for body mass index, ethnicity, meniscal injury, graft choice, contact sport, activity level, and preoperative Lysholm and Tegner scores. IKDC, International Knee Documentation Committee subjective knee evaluation form.

b College or more.

Graft choice (autograft or allograft), meniscal lesions at the time of surgery, injury mechanism, competition level, and medial collateral ligament or lateral collateral ligament pathology did not predict change in IKDC score from baseline to last follow-up.

### Return to Sport

Questionnaires regarding knee stability and return to sport were returned by 731 (35.8%) patients at a mean follow-up of 8.1 years (range, 6.7-9.8 years). In total, 633 (87%) patients reported that the knee felt stable in daily or athletic activities, whereas 506 (69%) patients reported that they had returned to sport. The most common reasons for not returning to sport were fear of new injury and change in lifestyle (Table 6).

**Table 6:** Subjective Knee Stability and Return to Sport as Reported by 731 Patients at a Mean Follow-up of 8.1 Years (Range, 6.7-9.8 Years) After Anterior Cruciate Ligament (ACL) Reconstruction

Questionnaire Item	n (%)
Does your knee feel unstable or give way during your daily life or athletic activities?	
Yes	98 (13.4)
No	633 (86.6)
Have you given up activities you enjoyed doing because of the knee?	
Yes	233 (31.9)
No	498 (68.1)
Have you returned to the sport you did before you ruptured your ACL, and if not, why?	
Yes	506 (69.2)
No	225 (30.8)
Afraid or worried it will happen again	74 (32.9)
Other interest/different life situation	60 (26.7)
Too painful	28 (12.4)
Don’t feel confident	15 (6.7)
Unable to return to same level	12 (5.3)
Knee feels unstable	12 (5.3)
ACL rerupture	8 (3.6)
Other injury	8 (3.6)
Other reason, unspecified	5 (2.2)
Was told to stop	3 (1.2)

### Revision ACL Surgery and Subsequent Nonrevision Knee Surgery

After a mean follow-up of 9 years (range, 8-11 years), 534 (26%) patients underwent at least 1 subsequent knee surgery, to either the same knee or the contralateral knee. This included 148 (7.2%) patients who underwent revision ACLR (Table 7), 270 (13.2%) patients who underwent nonrevision knee surgery to the ipsilateral knee, and 116 (5.7%) patients who underwent primary ACLR of the contralateral knee. The most common procedures were meniscal surgery (141 patients; 6.9%) and arthroscopic debridement (79 patients; 3.9%) (Appendix Table A2, available online). Knee arthroplasty had been performed in 4 (0.2%) patients. These patients were aged 42, 43, 44, and 47 years at the time of ACLR and underwent knee arthroplasty at a mean age of 54 years.

**Table 7:** Rate of Revision Within 9 Years (Range, 8-11 Years) of Primary Anterior Cruciate Ligament Reconstruction (ACLR)^*a*^

	ACLR Revision		
	No	Yes	*P* Value
Overall (N = 2042)	1894 (92.8)	148 (7.2)	
Sex			
Male (n = 1172)	1083 (92.4)	89 (7.6)	}.48
Female (n = 870)	811 (93.2)	59 (6.8)	
Age, y, mean (95% CI)	30.2 (29.6-30.7)	23.5 (21.9-25.2)	<.001
Graft choice (n = 1695)^*b*^			
Autograft (n = 1295)	1191 (92.0)	104 (8.0)	
Bone–patellar tendon–bone (n = 797)	734 (92.1)	63 (7.9)	} .92
Hamstring (n = 496)	456 (91. 9)	40 (8.1)	
Quadriceps (n = 2)	1	1	
Allograft (n = 400)	381 (95.3)	19 (4.7)	
Achilles tendon (n = 312)	296	16	.027^*c*^
Hamstring (n = 31)	29	1	
Quadriceps bone (n = 7)	7	0	
Unspecified (n = 4)	4	1	
Tibialis tendon (n = 46)	45	1	
No meniscal tear (n = 1024)	933 (91.1)	91 (8.9)	} .004
Meniscal tear (n = 1018)	961 (94.4)	57 (5.6)	
Medial (n = 360)	339 (94.2)	21 (5.8)	
Lateral (n = 417)	389 (93.3)	28 (6.7)	
Both (n = 241)	233 (96.7)	8 (3.3)	
Meniscal treatment			
Meniscectomy (n = 498)	480	18 (3.6)	} .003
Meniscal repair (n = 318)	291	27 (9.3)	
Cartilage injury (n = 1680)			
Yes (n = 415)	398 (95.9)	17 (4.1)	} .004
No (n = 1265)	1160 (91.7)	105 (8.3)	

a Data are expressed as n (%) unless otherwise noted. A total of 15 patients underwent 2 revisions, and 1 patient underwent 3 revisions.

b Data were missing for 347 (17%) patients.

c All autograft compared with all allograft.

**Figure 2.**
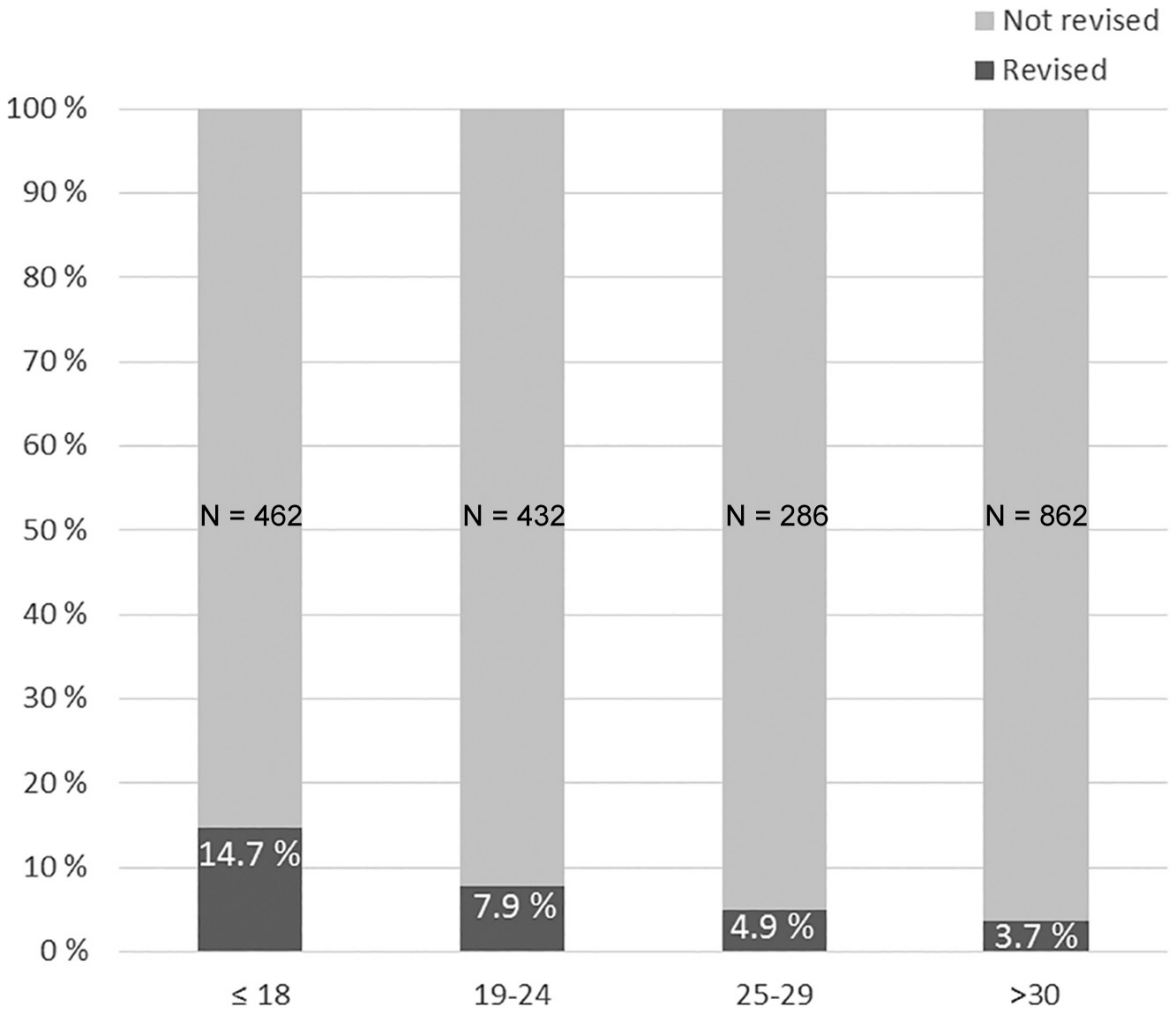
Revision rates at 9 years (range, 8-11 years) after primary anterior cruciate ligament reconstruction by age groups. Revision numbers (R) in each age group: ≤18 years, R = 68 (14.7%); 19-24 years, R = 34 (7.9%); 25-29 years, R = 14 (4.9%); >30 years, R = 32 (3.7%).

Age was a notable risk factor for revision. Nearly 15% of patients 18 years or younger had undergone revision, compared with <4% of patients 30 years or older (*P* < .0001) (Figure 2). Patients with isolated lateral meniscal tears had a higher risk of revision if the meniscus was repaired (12/115; 10.4%) compared with if it was treated with meniscectomy (10/243; 4.1%; *P* = .02). However, this was confounded by age. Patients undergoing lateral meniscal repair were younger than patients having a lateral partial meniscectomy (22.8 [95% CI, 21.1-24.5] vs 28.2 [95% CI, 26.8-29.6] years, respectively; *P* < .001). Patients who underwent meniscal repair were significantly younger than patients who underwent meniscectomy (24.7 [95% CI, 23.5-25.8] vs 33.1 [95% CI, 31.9-34.2] years, respectively; *P* < .001).

When we controlled for confounding factors, the statistically significant risk factors for revision were age, sex, meniscal injury at the time of reconstruction, and Marx score at baseline (Table 8). Although cartilage lesion size and education level were strong predictors for PROM results at final follow-up, neither affected revision risk. Ethnicity, body mass index, and sport type were also unrelated to revision risk.

**Table 8:** Multivariate Backward Stepwise Logistic Regression Analysis Presenting Risk Factors for Revision Surgery^*a*^

Variable	Odds Ratio (95% CI)	*P* Value
Age	0.95 (0.93-0.97)	<.001
Male sex	1.5 (1.04-2.22)	.031
No meniscal tear	1.76 (1.23-2.53)	.02
Marx score (baseline)	1.05 (1.00-1.10)	.037
Body mass index	0.95 (0.90-1.01)	.088

a Adjusted for graft choice (allograft vs autograft), ethnicity (White vs non-White), smoking status, level of education, contact sport, cartilage lesions, baseline patient-reported outcome measures (International Knee Documentation Committee subjective knee evaluation form, Tegner, Lysholm). Complete data set available for 1884 (92.3%) of 2042 patients entered in analysis, with 141 revisions.

## Discussion

The main finding in this study was that the postoperative improvements in IKDC scores were sustained 7 years after ACLR for the majority of patients. The strongest predictor for poor IKDC score at final follow-up was a cartilage lesion >2 cm^2^ identified during ACLR. Furthermore, nearly 70% of patients had returned to sport after 8 years, and 87% believed that their knee was stable in daily and athletic activities. The overall revision rate was 7%. In total, 26% of patients needed subsequent knee surgery to either the ipsilateral or the contralateral knee within 9 years of ACLR. Young male patients had better patient-reported outcomes but also a higher risk of revision surgery.

Our findings are consistent with outcomes reported by the MOON group, who also reported sustained improvements in IKDC scores after 10 years.^19^ They noted that the Marx activity score declined, despite sustained high IKDC and Knee injury and Osteoarthritis Outcome Score (KOOS) scores, reflecting the natural decline in athletic participation as patients become older, rather than the outcome of the ACLR per se. This is supported by our results, with a high proportion of patients who had a subjective feeling of a stable knee, despite the decline in Marx score over time. Furthermore, the youngest patients had the highest Marx score at baseline but also the largest decline over time, so that the differences between the age groups were not present 8 years later.

### Predictors for IKDC

The main risk factor for a poor IKDC score 7 years after ACLR was a cartilage lesion >2 cm^2^ at the time of surgery. This is in line with a study of >8000 patients from the Norwegian and Swedish ACL registries.^27,30^ Patients with concomitant full-thickness cartilage lesions reported worse outcome in all of the KOOS subscales compared with patients without cartilage lesions 2 years after ACLR. In the United States, the MOON group also confirmed that cartilage lesions at the time of surgery were associated with lower PROM scores at 10 years.^19^

A meniscal lesion identified at the time of ACLR was not a predictor for poor IKDC outcome after 7 years. This result is mirrored by a report from the Norwegian and Swedish ACL registries that did not find meniscal injuries to be associated with worse PROM scores.^27^ The MOON group further corroborated this, although they did find that lateral meniscal tears had a small negative effect of the KOOS Quality of Life subscale score at 10 years.^19^ However, the same group reported that a small, untreated tear of the lateral meniscus was predictive of better PROM scores at 6 years compared with a normal lateral meniscus.^6^ Furthermore, patients who underwent lateral meniscal excisions did better than patients with no tears. These somewhat counterintuitive results were explained by the altered kinematics of the knee after ACLR, with altered loading of the lateral compartment.^12,20^ Difference in activity level between the groups could also partly explain the results. Another possible explanation is that patients with injury to the meniscus had less concomitant injury to the articular cartilage, due to a mechanism of injury that loaded the meniscus rather than compressive loading of the joint surface.^6^ We speculate that an injury mechanism that primarily loads the meniscus rather than the articular cartilage surface is less detrimental to knee function. Further study would be necessary to test this hypothesis. Lateral meniscal tears did not affect the IKDC result in our study. Given these results, it seems that the presence of meniscal tears at the time of ACLR does not influence PROMs within the first 10 years after surgery, at least not to any substantial degree. This is useful information to guide patient expectations.

### Return to Sport

Our group demonstrated a return-to-sport rate of 87% in athletes at 2 years of follow-up.^22^ The present study included a variety of athletic patients and provides prognostic 8-year follow-up for return to sport for the average patient, not only high-performing athletes. We found a return-to-sport rate of nearly 70%, which is slightly less than the overall return-to-sport rate of 73% reported in a recent systematic review.^8^ However, we had a much longer follow-up of 8 years, compared with the mean follow-up of 3.4 years in the 20 publications included in the review. Our study found that fear of new injury or change in life situation or interest were the most common reasons for not returning to sport. This is almost identical to a report from Ireland, which found that failure to return to sport was due to external life and psychological factors associated with their injury rather than the outcome of the surgery itself.^29^ Considering the natural decline in athletic participation over time, we consider the return-to-sport rate of nearly 70% after 8 years to be an excellent result.

### Predictors for Revision

The revision rate was 7.2% after a mean follow-up of 9 years, which is comparable with the revision rate of 7.7% after 6 years reported by the MOON group.^11^ Like others, we identified age as a strong predictor for revision.^11,14,16,24^ It is well known that female patients have a higher risk for primary ACL injury,^9,18^ and historically female patients have also been considered to have a higher revision risk.^14^ However, more recent reports have not been able to identify sex as a predictor for revision surgery, suggesting that the increased risk for rupture of the native ACL in women is not transferred to the surgically reconstructed ACL.^7,11,24^ In contrast, we found that male sex was a predictor for revision. Our institution treats high-performing young male athletes who return to sport earlier and partake in more knee-demanding sports, which may explain these findings. Our results are supported by a large study from Kaiser Permanente that also found an increased risk of revision in male patients younger than 21 years.^16^

Patients with a meniscal tear identified during the index surgery were less likely to undergo ACLR revision during the follow-up period of 9 years. This was somewhat surprising to us, because Parkinson et al^23^ found meniscal deficiency to be a risk factor for revision. They analyzed risk factors for graft failure in 118 patients after 2 years, of whom 20 patients were considered to have experienced failure. The authors concluded that meniscal deficiency was the most important factor for ACL graft failure. However, the study was underpowered, with only 2-year follow-up. In a recent large study from Kaiser Permanente with 12 years of follow-up, meniscectomy did not influence revision rates.^32^ Vindfeld et al^31^ conducted a matched case-control study of 100 patients undergoing revision ACL surgery and 100 matched controls who were not revised at 11-year follow-up. Like us, those investigators found that patients who did not undergo revision were more likely to have had a meniscal tear identified during the primary surgery. The patients who underwent revision were more likely to have had a failed meniscal repair, but the authors pointed out that this might be caused by the instability of the knee joint due to the graft failure rather than the failed meniscal repair causing the graft to rupture. We also found that patients treated with meniscal repair had a higher risk of revision compared with patients treated with meniscectomy, but this was confounded by a significant age difference of 9 years. The patients treated with meniscal repair were younger patients (mean age 24.7 years) returning to high-risk athletic activities, which explains the high revision risk, whereas the patients who underwent meniscectomy were on average 33.1 years old at the time of ACLR, which is an age group with an established low revision risk.

### Limitations

The study was conducted in a single institution, which may reduce external generalizability, and there were 883 patients who were not included, which may constitute a selection bias. Although every effort was made to retrieve information about revision surgery, some patients may have been treated at other institutions without our knowledge. Although 69% of patients reported that they had returned to the sport they did before the injury, we do not know whether they returned to the same level of play. There was a sizable loss to follow-up, which introduces selection bias. However, we have conducted an in-depth analysis of nonresponders and have not found significant differences in outcome or revision rates between responders and nonresponders.^26^ Furthermore, cartilage lesions were assessed only by size, not depth, and our study did not have enough power to analyze the effect of the location in the knee joint of the cartilage lesions.

## Conclusion

Our study provides realistic expectations of medium-term outcome after ACLR as well as predictors for poor patient-reported outcomes and revision rates. After a mean follow-up of 7 years, patient-reported outcomes remained good to excellent for the majority of patients. A cartilage lesion >2 cm^2^ at the time of surgery and low educational level were the strongest predictors for poor outcome. After 8 years, about 70% of patients had returned to sport, and 87% of patients believed that their knee was stable in daily and athletic activities. Young male patients had better patient-reported outcome but also a higher risk of revision. We found a 7% risk of ACLR revision and 13% risk of ipsilateral nonrevision knee surgery within 9 years. The total risk of subsequent knee surgery within 9 years of ACLR was 26%, emphasizing the significant implications of an ACL tear.

## Supplemental Material

sj-pdf-1-ajs-10.1177_03635465211060333 – Supplemental material for Patient-Reported Outcome, Return to Sport, and Revision Rates 7-9 Years After Anterior Cruciate Ligament Reconstruction: Results From a Cohort of 2042 Patients Click here for additional data file. Supplemental material, sj-pdf-1-ajs-10.1177_03635465211060333 for Patient-Reported Outcome, Return to Sport, and Revision Rates 7-9 Years After Anterior Cruciate Ligament Reconstruction: Results From a Cohort of 2042 Patients by Per-Henrik Randsborg, Nicholas Cepeda, Dakota Adamec, Scott A. Rodeo, Anil Ranawat and Andrew D. Pearle in The American Journal of Sports Medicine
