# Supplementary material for: Patient-Reported Outcome, Return to Sport, and Revision Rates 7-9 Years After Anterior Cruciate Ligament Reconstruction: Results From a Cohort of 2042 Patients
Source: Am J Sports Med. 2022 Jan 18;50(2):423–32. doi: 10.1177/03635465211060333 (PMC8829731; doi:10.1177/03635465211060333)
Supplement: sj-pdf-1-ajs-10.1177_03635465211060333 – Supplemental material for Patient-Reported Outcome, Return to Sport, and Revision Rates 7-9 Years After Anterior Cruciate Ligament Reconstruction: Results From a Cohort of 2042 Patients [file sj-pdf-1-ajs-10.1177_03635465211060333.pdf]

| **Variable** | Univariate regression coefficient (95 % CI) | p-value |
|---|---|---|
| Baseline IKDC | -0.93 (-0.99 – 0.88) | <0.001 |
| Baseline Marx | 0.22 (0.05 – 0.40) | 0.014 |
| Age | -0.03 (-1.11 – 0.041) | 0.4 |
| Sex | | |
| Male (ref) | REF | |
| Female | -1.36 (-3.12 – 0.40) | 0.13 |
| BMI | -0.21 (-0.45 – 0.029) | 0.085 |
| Smoker | | |
| No (ref) | REF | |
| Yes | -3.13 (-6.42 – 0.15) | 0.061 |
| Level of Education | | |
| High School or less | REF | |
| College or more | 2.07 (0.16 – 3.99) | 0.034 |
| Race | | |
| White | REF | |
| Non-White | -0.62 (-2.80 – 1.57) | 0.58 |
| Level of Activity | | |
| Recreational | REF | |
| High School | -0.37 (-3.04 – 2.30) | 0.79 |
| College | -1.16 (-5.22 – 2.90) | 0.58 |
| Professional | -2.83 (-13.27 – 7.61) | 0.59 |
| Non-Contact Sport | REF | |
| Contact Sport | 0.086 (-2.45 – 2.63) | 0.95 |
| Low-Risk sport | REF | |
| High-Risk Sport* | 0.60 (-1.16 – 2.37) | 0.50 |
| Meniscus Injury | | |
| No | REF | |
| Yes | 0.017 (-1.74 – 1.78) | 0.99 |
| Meniscal treatment | | |
| Repair | REF | |
| Meniscectomy | 2.12 (-0.77 – 5.01) | 0.15 |
| Cartilage Injury | | |
| No | REF | |
| Yes | -0.38 (-2.50 – 1.74) | 0.73 |
| Cartilage lesion size | | |
| $<2$ cm$^2$ | REF | |
| $\geq 2$ cm$^2$ | -15.0 (-24.45 - -5.65) | 0.02 |
| Autograft | REF | |
| Allograft | -1.69 (-3.83 – 0.46) | 0.12 |

**Appendix Table A1**: Univariate Regression analysis to identify predictors for change in IKDC score from baseline to final follow-up. Each predictor is adjusted for baseline IKDC only.

CI Confidence Interval, BMI Body Mass Index, IKDC International Knee Documentation Committee subjective knee evaluation form, REF reference variable. *Basketball, Soccer, Football, LaCrosse, Ski, Volleyball

| Subsequent non-revision ipsilateral knee surgery | First subsequent surgery | Second subsequent surgery | Third subsequent surgery |
|---|---|---|---|
| Overall | 270 (13.2 %) | 30 (1.5 %) | 8 (0.4 %) |
| Meniscal surgery | 141 (6.9 %) | 14 (0.7 %) | 2 (0.1 %) |
| Debridement/lose bodies | 79 (3.9 %) | 11 (0.5 %) | 3 (0.1 %) |
| Removal of hardware | 29 (1.4 %) | 1 (0.05 %) | 1 (0.05 %) |
| Cartilage surgery | 8 (0.4 %) | 3 (0.1 %) | 1 (0.05 %) |
| Other ligament surgery | 4 (0.2 %) | - | - |
| Arthroplasty | 4 (0.2 %) | - | - |
| MUA | 1 (0.05 %) | 1 (0.05 %) | 1 (0.05 %) |
| Other | 4 (0.2 %) | - | - |

**Appendix Table A2:** Percentage of patients that underwent subsequent ipsilateral non-revision knee surgery within 9 (range 8-11) years of primary Anterior Cruciate Ligament reconstruction. MUA Manipulation Under Anesthesia.
